# Comments on “Reflections on the Inclusion of Direct-Care Physicians as Educators in Community Hospitals”

**DOI:** 10.1590/0100-6991e-20243859-en

**Published:** 2024-12-02

**Authors:** ALEXANDRE SLULLITEL

**Affiliations:** 1 - Universidade de São Paulo, Anestesiologia - São Paulo - SP - Brasil

**Keywords:** Medical Education, Preceptorship, Health Services Needs and Demands, Learning, Educação Médica, Preceptoria, Necessidades e Demandas de Serviços de Saúde, Aprendizagem

## Abstract

We discuss the arguments exposed in the Letter to the Editor “Reflections on the Inclusion of Direct-Care Physicians as Educators in Community Hospitals”, exploring the teaching competencies necessary for community preceptors in the context of medical education, highlighting the growing responsibility of these professionals in the training of future physicians in health-deprived regions. From a narrative review, we analyze faculty development (FD) programs, emphasizing their importance in improving teaching skills, creating support networks, and providing personalized content for specific challenges. Among the competence domains identified are teaching skills, evaluation criteria, professionalism, communication, and leadership/management. In addition, we suggest Entrustable Professional Activities (EPA) as tools to assess and develop these competencies. FD programs, when well structured, benefit preceptors, students, and communities, improving the quality of teaching and care outcomes.

## RESUMO

São debatidos os argumentos expostos na Carta ao Editor “Reflexões sobre a Inserção de Médicos Assistenciais como Educadores em Hospitais Comunitários”, explorando as competências docentes necessárias para preceptores comunitários no contexto da educação médica, destacando a crescente responsabilidade desses profissionais na formação de futuros médicos em regiões carentes de saúde. A partir de uma revisão narrativa, são analisados programas de desenvolvimento do corpo docente (Faculty Development - FD), enfatizando sua importância para aprimorar habilidades de ensino, criar redes de apoio e fornecer conteúdos personalizados para desafios específicos. Entre os domínios de competência identificados estão habilidades de ensino, critérios de avaliação, profissionalismo, comunicação e liderança/gestão. Além disso, são sugeridas atividades práticas profissionais (EPA) como ferramentas para avaliar e desenvolver essas competências. Os programas de FD, quando bem estruturados, beneficiam preceptores, alunos e comunidades, melhorando a qualidade do ensino e dos desfechos assistenciais.

## INTRODUCTION

There is no doubt that community physicians have gradually assumed the responsibility of preceptorship in primary care both nationally and internationally, especially in regions with a shortage of health professionals. The situation accelerated from 2010 onwards, with the formulation of government public policies that sought to stimulate the unbalanced training of medical preceptors without the respective proportionality of expansion of programs for the development of faculty. Such policies coincide with the proliferation of medical schools that use direct care health professionals without pedagogical skills to fulfill teaching functions, such as preceptors. To this end, it is necessary to question what would be the competencies necessary to perform these functions and what are the possible results of such experiences in the learning model.

A narrative review analyzed faculty development programs designed specifically for community preceptors with the goal of identifying how such programs can support medical schools to improve preceptors’ training and effectiveness^1^. The review highlights the importance of community preceptors in medical education, as they play a crucial role in the training of future health professionals.

Several FD programs that have been implemented and their potential benefits for preceptors and medical students are analyzed. The authors emphasize the need for customized development programs that address the unique challenges faced by community preceptors.

What would be the possible implications for medical schools? By investing in faculty development for community preceptors, medical schools can improve the quality of education provided to students and enhance the overall training experience.

 Some strengths of FD programs that deserve to be highlighted are:

1) Improved teaching skills: FD programs can significantly improve the teaching skills of community preceptors, leading to better educational experiences for medical students.
2) Support network: These programs often create a community among preceptors, promoting collaboration and sharing best practices.
3) Personalized content: Effective FD programs are designed to address the specific challenges faced by community preceptors, making training more relevant and applicable.
4) Increased engagement: Preceptors who participate in FD programs often report higher levels of engagement and satisfaction in their teaching roles.

On the other hand, there may also be points of weakness in these programs, among which it is worth highlighting:

1) Resource limitations: Many community preceptors may not have access to adequate resources or time to fully participate in FD programs, which can limit their effectiveness.
2) Variability in Implementation: The quality and structure of FD programs can vary widely, leading to inconsistent experiences for preceptors.
3) Resistance to Change: Some preceptors may be resistant to adopting new teaching methods or practices introduced into FD programs, which can hinder their success.
4) Limited Evaluation: There is often a lack of robust evaluation mechanisms to assess the long-term impact of FD programs on preceptors and students.

These insights highlight the importance of carefully designing and implementing FD programs to maximize their benefits while addressing potential challenges.

Still in the same sense, what would be the competencies in need of development in such FD programs?

An article entitled “Teaching Competencies for Community Preceptors”, published in Family Medicine, describes essential competencies for community preceptors involved in medical education^2^. It presents a framework consisting of five competency domains with 21 associated sub-competencies, designed to assess the needs of preceptors, support their development, and assess their effectiveness in teaching roles. The main areas of competence include the following:

1) Teaching Skills: This domain focuses on the ability to effectively impart knowledge and skills to students while ensuring that teaching methods are engaging and appropriate for the audience.
2) Evaluation criteria: Preceptors should be qualified to evaluate students’ performance and provide constructive feedback to facilitate their growth.
3) Professionalism: This includes demonstrating ethical behavior, maintaining confidentiality, and serving as a role model for students.
4) Communication: Effective communication skills are essential for interacting with students, colleagues, and patients, fostering a positive learning environment.
5) Leadership and Management: Preceptors must exhibit leadership qualities and be able to manage educational activities, including organizing learning experiences and mentoring students.

From the definition of such competencies, Entrustable Professional Activities (EPA) could be elaborated to formulate the development and demonstration of such competencies^3^. From the above-mentioned competences, we could draw up an EPA corresponding to each specific domain, for example:

- Teaching Skills - Conduct a teaching session in small groups.
- Assessment - Provide feedback on a student’s clinical performance.
- Professionalism - Demonstrate ethical decision-making in patient care scenarios.
- Communication - Communicating effectively with patients and students during clinical encounters.
- Leadership and management - Organizing and leading a clinical teaching session.

Subsequently, it would be necessary to define evaluation methods and criteria, create models for training and development, and implement the respective EPAs in the training program with continuous effectiveness evaluation.

## CONCLUSION

Direct care physicians invested in the role of educators offer several benefits, particularly in improving patient satisfaction, enhancing medical education, and fostering a favorable learning environment for future health care professionals.

Such physicians can become effective teachers, especially when they are confident in their teaching abilities and actively participate in well-designed faculty development programs. These programs should aim to help improve their teaching skills and to positively influence their attitudes towards teaching, ultimately benefiting both the clinicians themselves and their students, and improving care outcomes for the community.
